# Archaea as a Model System for Molecular Biology and Biotechnology

**DOI:** 10.3390/biom13010114

**Published:** 2023-01-06

**Authors:** Federica De Lise, Roberta Iacono, Marco Moracci, Andrea Strazzulli, Beatrice Cobucci-Ponzano

**Affiliations:** 1 Institute of Biosciences and BioResources, National Research Council of Italy, Via P. Castellino 111, 80131 Naples, Italy; 2 Department of Biology, University of Naples Federico II, Complesso Universitario di Monte S. Angelo, Via Cinthia 21, 80126 Naples, Italy; 3 Task Force on Microbiome Studies, University of Naples Federico II, 80100 Naples, Italy; 4 NBFC, National Biodiversity Future Center, 90133 Palermo, Italy

**Keywords:** archaea, hyperthermophiles, recoding, molecular biology, CAZymes, metagenomic

## Abstract

Archaea represents the third domain of life, displaying a closer relationship with eukaryotes than bacteria. These microorganisms are valuable model systems for molecular biology and biotechnology. In fact, nowadays, methanogens, halophiles, thermophilic euryarchaeota, and crenarchaeota are the four groups of archaea for which genetic systems have been well established, making them suitable as model systems and allowing for the increasing study of archaeal genes’ functions. Furthermore, thermophiles are used to explore several aspects of archaeal biology, such as stress responses, DNA replication and repair, transcription, translation and its regulation mechanisms, CRISPR systems, and carbon and energy metabolism. Extremophilic archaea also represent a valuable source of new biomolecules for biological and biotechnological applications, and there is growing interest in the development of engineered strains. In this review, we report on some of the most important aspects of the use of archaea as a model system for genetic evolution, the development of genetic tools, and their application for the elucidation of the basal molecular mechanisms in this domain of life. Furthermore, an overview on the discovery of new enzymes of biotechnological interest from archaea thriving in extreme environments is reported.

## 1. Introduction

The distinctiveness of archaea became apparent in the late 1970s when Carl Woese and his colleagues used the small subunit rRNA as a molecular marker [1,2]. The phylogenetic tree suggested a closer relationship between archaea and eukaryotes than between bacteria, and genome sequencing in the 1990s confirmed that archaea are a genetic mosaic; their information processing systems show significant homology with their eukaryotic counterparts, while most of the basal functions are bacterial-like [3]. The link between the archaeal and eukaryotic domains has been reinforced in light of the recent discovery of novel archaeal lineages, termed “ASGARD”, comprising the species Lokiarchaeota and Thorarchaeota. It has been hypothesized that eukaryotes are descended from an archaeal species related to the ASGARD, as a large number of gene families previously thought to be unique to Eukarya are present in ASGARD genomes. This fosters the interest in the studies on fundamental processes in archaea to shed light on the evolution of life [4]. Nowadays, methanogens, halophiles, thermophilic euryarchaeota, and crenarchaeota are the four groups of archaea for which genetic systems have been well established, making them suitable as model systems and allowing the increasingly sophisticated study of archaeal gene functions. For example, methanogens are used as models for archaeal replication, transcription, osmoregulation, and protein structure [4]. Furthermore, thermophiles are used to explore a variety of aspects of archaeal biology, including cellular responses to stress, CRISPR systems, DNA replication and repair, transcription and its regulation, and carbon and energy metabolism [5]. Moreover, extremophilic archaea nowadays represent a potentially valuable source for biological and biotechnological applications, and there is growing interest in their potential applications in molecular biology and in the development of engineered strains [6]. In fact, as they can perform microbial processes under extremely hostile conditions, they can act as a source of stable enzymes and biomaterials [7,8,9].

In this review we report on some of the most important aspects of the use of archaea as a model system for genetic evolution, the development of genetic tools, and their application for the elucidation of the basal molecular mechanisms in this domain of life. Furthermore, an overview on the discovery of new enzymes with a potential biotechnological application from archaea thriving in extreme environments is also reported.

## 2. Archaea as a Model System for Genetic Evolution

From the perspectives of molecular and evolutionary biology, archaea are fascinating organisms to study. Archaeal cells are unicellular and lack a nucleus, and therefore, they resemble bacterial cells in terms of ultrastructure. However, the molecular machineries of archaea are similar to those present in eukaryotes [10]. For instance, the proteins involved in DNA replication, transcription, and translation are quite similar to the eukaryotic ones. For these reasons, early research on archaea was largely motivated by the quest to understand more basic model systems for eukarya. For instance, the study of the archaeal enzymes involved in DNA replication and repair has taught us a lot about their eukaryotic counterparts. The lack of genetic tools and the difficulties in cultivating archaea in labs has long hindered their study. The development of genetic tools in several archaeal species over the past decade has accelerated our understanding of enzymes and metabolic pathways and enabled more detailed analysis of basic cellular processes. This allows the emergence of archaea as a eukaryal model and has also provided insights into diversity in this domain. In fact, species within a phylum showed substantially different strategies for basic cellular mechanisms. The ever-expanding arsenal of genetic and biochemical tools of *H. volcanii*, for example, solidifies its status as model organism for understanding the evolutionary history of DNA replication and repair and for discovering what is common to eukaryotes and what is exclusive to archaea [11].

Nowadays, different species from methanogenic, halophilic, thermophilic euryarchaeota, and crenarchaeota have well established genetic systems, making them suitable as model systems and allowing for the sophisticated study of archaeal gene functions.

### 2.1. Genetic Tools

Genetic manipulation is considered a crucial tool for the understanding of gene functions. The traditional strategy in archaea to identify groups of genes involved in a specific biological function is random mutagenesis, followed by the isolation of mutant strains and screening of the genes that complement the mutation [12]. In the last decades, the development of genetic tools enables, in vivo, the introduction, modification, or deletion of a specific gene of interest, allowing for understanding the importance, or lack thereof, of a specific gene [13]. The development of genetic manipulation systems in archaea lagged behind compared to bacterial ones, first of all because of the difficulties in developing efficient selectable markers [14]. In fact, in archaea, the majority of bacterial antibiotics used as selection markers are ineffective. In addition, for extremophilic archaea, thriving at conditions very dissimilar from those of the mesophilic bacteria made it difficult to develop appropriate screening methods.

In more recent years, the number of archaeal genetic tools has increased in several species from the phyla Euryarchaeota and Crenarchaeota [12]. Among them, genetic systems have been established for *Thermococcus kodakaraensis*, using mutant host strains in which the *purF* and/or *trpE* gene were deleted, for *Haloferax volcanii*, where a system for protein overexpression, inducible promoters, a gateway system for deletion construction, and several reporter genes have been produced in the past years [15], and *Pyrococcus furiosus*, for which genetic selections and counterselections based on uracil biosynthesis lead to the achievement of single- and double-deletion mutants of the two gene clusters that encode the two cytoplasmic hydrogenases [16].

In addition, significant progress has been made in the evolution of plasmid systems, and the implementation of protocols, such as DNA recombination techniques and the use of a selective and integrative shuttle vector as reporter gene, for the production of deletion mutants for several *Sulfolobus* and *Saccharolobus* species have been achieved [14]. As stated above, the major impediment to the evolution of techniques for the directed gene deletion in Sulfolobaceae has been the scarcity of appropriate selectable markers; in fact, despite the identification of uracil producers’ auxotrophic strains, several years before both in *Sulfolobus acidocaldarius* [17] and *Saccharolobus solfataricus* [18], they have not been employed as selective markers, as the presence of uracil traces, in the Gelrite polymer solid media, caused a quite high background level. A more serious issue was that *S. solfataricus* strains P1 and P2, the strains commonly utilized, do not perform the recombination of foreign DNA plasmid into their chromosome [14]. Later, it was discovered that the PBL2025 strain, a natural mutant of *S. solfataricus* 98/2, is capable of homologous recombination; this strain was utilized to produce deletion mutants. In this strain, the *lacS* gene, encoding for a beta-galactosidase allowing the growth of lactose as carbon source, is missing and was employed as a selection marker [14,19]. A detailed description of the archaeal genetic tools goes beyond the aims of this review. In the next paragraphs, the most relevant genetic tools for hyperthermophiles that have improved our understanding of gene function will be described, with an emphasis on transformation and gene knockout techniques. Furthermore, in Section 2.2, some of the most relevant applications of the developed genetic tools that have allowed for significant progress in the study of the basic molecular mechanisms of archaea will be described.

#### 2.1.1. Transformation, Heterologous Protein Expression and Genetic Manipulation

The limitation in the heterologous expression of archaeal and hyperthermophilic archaeal proteins is that they often cannot be functionally produced in mesophilic bacterial hosts [20]. This can be for several reasons e.g., biased codon use, archaeal expression signals, the need for particular polypeptides that are involved in processing or assembly, post-translational modifications, or a unique cellular environment for proper expression and folding, which cannot be found in non-archaeal cells. For this purpose, the past years have seen significant progress in archaeal genetic investigations, and a lot of work has gone into developing archaeal expression systems [21]. In this context, *halophilic* archaea have been established as a good controlled and inducible expression system, even if the near-saturating intracellular salinities usually damage downstream protein processing of non-halophilic proteins and frequently affect their folding [20]. In addition, a variety of methanogenic archaeal lineages have been modified to offer platforms for inducible and regulated protein recombinant expression [22], but none have been developed at high scale. Thermophilic and hyperthermophilic archaea, in particular, Thermococcales and Sulfolobales, have gained much attention due to their advantages in being versatile platforms for the recombinant expression of thermostable proteins that are very useful for a variety of biotechnological applications [23,24]. Among Thermococcales, *Thermococcus kodakarensis* has emerged as a suitable model species for the study of biological processes at high temperatures and as a platform for the recombinant expression of thermostable enzymes [25]. The creation of *T. kodakarensis* genomic and ectopic manipulation techniques that are extremely reproducible and accurate allowed for rational and iterative strain construction, controlled protein overexpression [20], and the introduction of protein tags or epitopes [26] that facilitate simple and easy protein purifications.

For example, it was demonstrated that positioning genes under the control of strong, constitutive promoters, such as those of the glutamate dehydrogenase gene (*TK1431*) or the cell surface glycoprotein gene (*TK0895*), the 3-hydroxy-3-methylglutaryl coenzyme A (*HMG-CoA*) reductase genes from *T. kodakarensis* and *Pyrococcus furiosus*, the endogenous pantoate kinase gene, and the α-1,4-glucan phosphorylase gene from *Saccharolobus solfataricus*, have been expressed in *T. kodakarensis*, leading to active proteins and also the possibility to use this strain for protein secretion using a signal peptide from *TK1675* [27].

Moreover, *T. kodakarensis* has also been used as a genetic tool platform to mutagenize genes in order to understand their physiological roles; for example, the role of specific transcription factors have been studied, including TFB1/2 and RNA polymerase subunit B (see below) [28,29,30], GAP:ferredoxin oxidoreductase (GAPOR), non-phosphorylating GAP dehydrogenase (GAPN), phosphorylating GAP dehydrogenase (GAPDH), glyceraldehyde 3-phosphate (GAP) and 3-phosphoglycerate (3-PGA) genes involved in glycolysis [31], and coenzyme A [32] and compatible solute biosynthesis, as the myo-inositol phosphate (DIP), which seems to plays a role in thermoprotection; in this context, mutant strains of *Thermococcus kodakarensis* altered in their capacity to synthesis DIP were created and used to investigate the role of DIP in the thermoadaptation of this microorganism; this was the first research employing mutants to show how compatible solutes play a role in the thermal adaptation of (hyper)thermophiles [33].

A difficulty encountered in having an efficient archaeal expression system is the availability of strains that can be easily transformed by exogenous DNA. *T. kodakarensis* is naturally competent and is able to integrate exogenous DNA in its genome through homologous recombination [34]. Instead, in *Pyrococcus furiosus*, two transformation strategies, based on shuttle vectors that replicate in both *P. furiosus* and *E. coli*, have been established. One is based on *P. abyssi’s* pYS2 shuttle vector system, used for the overexpression of the 3-hydroxy-3-methylglutaryl coenzyme A reductase in *P. furiosus*, in which selection is based on simvastatin resistance [35]. Another approach was based on the using of a variant isolated strain, named COM, which was particularly capable of taking in and recombining foreign DNA in both circular and linear forms; this strain was obtained by targeted gene disruption of the *pyrF* locus. With the use of this strain, single- and double-deletion mutants of the two putative operons that encode the two heterotetrameric cytoplasmic hydrogenases of *P. furiosus* have been produced, raising questions about their roles in *P. furiosus*’s metabolism [16].

Efficient host–vector systems as well as novel and conventional methods of genetic manipulation have been developed in crenarchaea Sulfolobales [30]. The genera *Sulfolobus* and *Saccharolobus* have a variety of naturally genetic elements, such as viruses, cryptic plasmids, and transposons, providing its unique potential for genetic application [30]. Several versatile genetic techniques have been developed for *S. acidocaldarius*, *S. islandicus*, and, to a lesser extent, also for *S. solfataricus* [24]. The first transformation system developed for Sulfolobales was pursued using the SSV1 virus for cells of the foreign host *S. solfataricus* P1 by electroporation [36]. After that, a restriction-deficient strain was produced, leading to significantly greater transformation rates for *S. acidocaldarius* [37] and demonstrating that, for Sulfolobus and Saccharolobus microorganisms, electroporation is a particularly effective method of transformation.

In *S. solfataricus*, as described above, the discovery of the natural mutant PBL2025, devoid of *lacS*, allowed for the complementation with transformed clones carrying an intact *lacS*, used asfor marker gene, based on their capacity of growth on a minimal medium that contains lactose; this method was,, for example used to create a mutant with the deletion of the α-amylase gene (*amyA*) in *S. solfataricus*, which lost the ability to grow on starch, glycogen, or pullulan as sole carbon and energy sources, not only clarifying the biological role of α-amylase, but also providing new methods for the genetic manipulation in *S. solfataricus* [38]. Among Sulfolobales, *S. acidocaldarius* is one of the most genetically tractable hyperthermophilic archaea, widely used for protein knockout and overexpression because of its genomic stability. The discovery of the maltose-inducible promoter of the putative maltose-binding protein in the *Saci*_1165 strain [39] and the identification of the uracil auxotrophic mutants, named MW001, allowed for developing a set of genetic tools, including several plasmids that can be used to create mutants with markerless deletions or to insert tags into the genome [30].

#### 2.1.2. Gene Knockout

Gene knockout is an important genetic tool for the study of gene functions. The first gene knockout performed in Sulfolobaceae was made using the host *S. solfataricus* PBL2002, a spontaneous mutant of the *S. solfataricus* 98/2 strain with an insertion in the *lacS* gene. This strategy was used for genetic studies of mercury resistance, the structure and function of flagella, sugar-binding proteins, copper-responsive expression, and the mechanism of DNA polymerase translation [40,41,42,43].

Another strategy of gene silencing was implemented in *T. kodakarensis*, based on the *pyrEF* gene and *pyrEF* selection [30]; this method was applied to *S. islandicus* E233, which carries a spontaneous deletion in *pyrEF* genes. In brief, a marker cassette was built with *pyrEF* as a marker gene and cloned into a *E. coli* plasmid. The transformation of *S. islandicus* cells led to the recombination between the target region and the marker gene cassette, replacing the target gene with *pyrEF*, allowing for the selection of knockout mutants [30]. This strategy was further implemented in order to obtain markerless mutants to make it possible to carry out other gene knockouts in the same strain. This purpose was pursued in *H. volcanii* by using three approaches: (i) the plasmid integration and segregation method, commonly known as the “pop-in and pop-out approach” (PIS) [44]; (ii) the marker replacement and looping out recombination (MRL); (iii) the Marker Insertion and target gene Deletion (MID) method.

**Figure 1:**
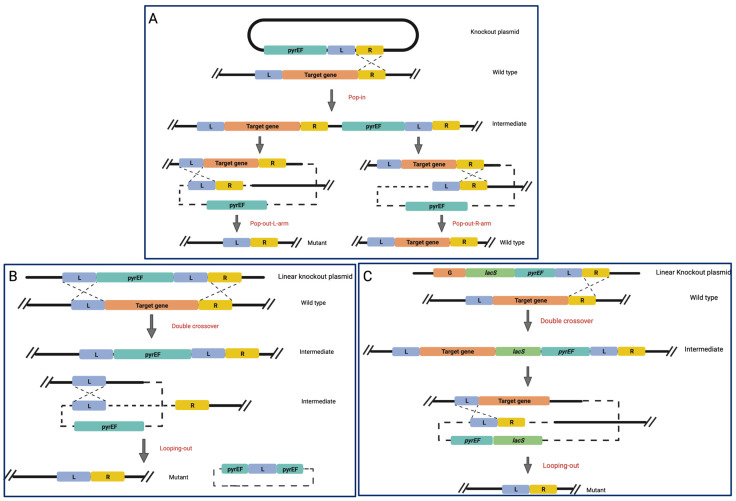
Schematic representation of three genome editing methods developed in *H. volcanii*. (**A**) Plasmid integration and segregation (PIS); (**B**) Marker replacement and looping out (MRL); (**C**) Marker insertion and target gene deletion (MID). In all panels, L and R arms are highlighted in blue and yellow, respectively; *lacS* and *pyrEF* genes markers are in green and teal, respectively. The *pyrEF* selection is used to illustrate the schemes, but any other efficient markers might be used [45]. This figure was created with BioRender.com (accessed on 7 December 2022).

In the “pop-in and pop-out approach” (PIS), schematized in Figure 1A, there are target gene’s left- and right-flanking arms (L and R arms) carried by the knockout plasmid. When the L and R arms are fused, a mutant gene allele is produced lacking some or all of the target gene’s coding sequence. Following transformation, the knockout vector is incorporated into one of the flanking arms of the target gene, resulting in transformants that carry a merodiploid form of the homologous sequence. The flanking arms are then allowed to segregate, resulting in the creation of the targeted knockout mutant or the original host (Figure 1A) [30,45].

In the marker replacement and looping out recombination (MRL) method, where one of the homologous sequence arms is repeated (Figure 1B), a linearized knockout plasmid containing redundant L-arm sequences is used. After transformation, the MRL plasmid and the chromosome underwent a two-fold crossover for the L-arms and R-arms, resulting in the merodiploid form of the L-arm in the chromosome of transformants able to grow on uracil-free plates. An MRL protocol was used to carry out experiments on several genes involved in DNA replication and repair, including those suspected of acting as replication clamps and initiators as well as those suspected of being involved in the base excision repair and nucleotide excision repair (NER) pathways [46].

Furthermore, in the so-called Marker Insertion and target gene Deletion (MID) approach (Figure 1C), three distinct sequence arms are used: a target gene arm, an L-arm, and an R-arm. As explained in Figure 1C, the knockout plasmid is arranged with a target gene arm, a selection marker, an L- and R-arm, and where the target gene arm is overlapping on a region of the target gene to be knocked out, favoring a double crossover in the target gene and R arm, determining the final deletion of the target gene and the marker cassette from the host chromosome by recombination. After the introduction of MID, loss-of-function analysis has been used to investigate the functions of several Sulfolobaceae genes, such as those associated with the NER pathway (see below) [47,48], the archaellum biosynthesis and function [49], and the synthesis of sulfoquinovose, a component of N-linked glycans on the surface-layer glycoprotein of *S. acidocaldarius* [30].

These systems have been used for several studies of essential genes in different archaeal species, especially to investigate the proteins involved in the duplication, transcription, and translation mechanisms; examples and details about the proteins studied are reported in the next paragraph.

**Figure 2:**
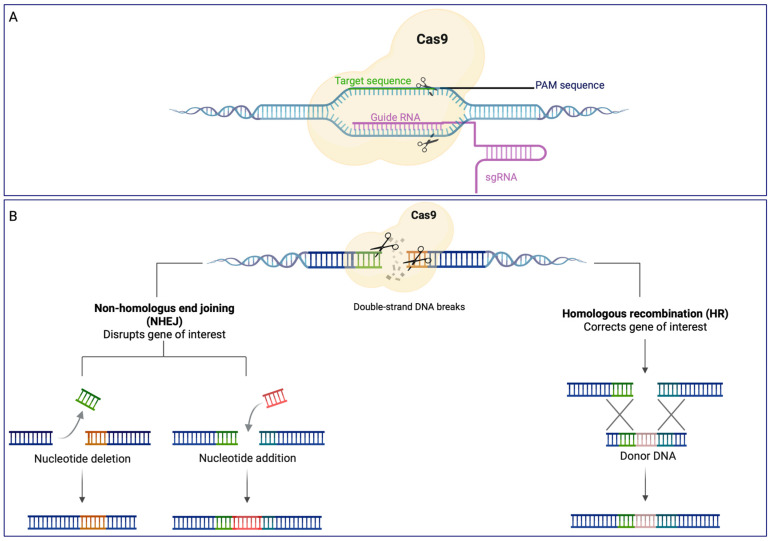
Cas9 endonuclease recruiting for target recognition using a single chimeric RNA (**A**) and genome editing (**B**). (**A**) When the targeted sequence (green) is immediately followed by a PAM sequence, a single chimeric guide RNA (violet) guides the active Cas9 endonuclease (light yellow) to cleave-site-specific DNA; (**B**) once double strand DNA breaks are performed, cells activate error-prone non homologous end-joining (NHEJ) repair pathways to fix the damage by adding random tiny insertions (red) or deletions at the cut spot; when a homologous DNA template is available, cells can repair their DNA through a process known as homologous recombination (HR), which leads to genomic knock-in (light brown) at the exact cut region. This figure was created with BioRender.com (accessed on 7 December 2022).

An efficient gene silencing tool that has gained a lot of attention in recent years is based on the CRISPR-Cas system (Clustered Regularly Interspaced Short Palindromic Repeats)-CRISPR associated) [50]. CRISPR-Cas is an adaptive immune system that protects from phages, viruses, and other foreign genetic elements [51]. It consists of CRISPR repeat spacer arrays that can be further transcribed into CRISPR RNA (crRNA) and trans-activating CRISPR RNA (tracrRNA), as well as a group of CRISPR-associated (cas) genes that encode Cas proteins with endonuclease activity. When foreign genetic elements invade prokaryotes, Cas proteins can break the invaders’ DNA into brief fragments, which are then incorporated into the CRISPR array as new spacers. When the same invader enters once more, crRNA will immediately identify it and bind with the foreign DNA, which directs Cas proteins to cleave foreign target sequences, thus defending the host [52]. The most studied CRISPR/Cas system is CRISPR/Cas9. RNA-guided Cas9 endonuclease and a single-guide RNA (sgRNA), a simplified form of tracrRNA and crRNA, are the two primary components of this system [53]; Cas9 and sgRNA form the Cas9 ribonucleoprotein (RNP) that, also thanks to a protospacer adjacent motif (PAM) sequence, can bind to and cleave the DNA target (Figure 2A). During the genome-editing process (Figure 2B) sgRNA recruits Cas9 to a specific site in the genome, leading to a double-stranded break (DSB). This break can be repaired by two endogenous self-repair mechanisms: the homology-directed repair (HDR) or the error-prone non-homologous end joining (NHEJ) pathways. The NHEJ process leads to random DNA base insertion/deletion mutations (indels) at the cut spot, determining changes in the target gene’s expression, including genetic knockdown. However, if a homologous DNA template is provided, cells perform homologous recombination (HR) as a repair strategy, leading to genomic knock-in at the specific cut site (Figure 2B).

Since its discovery, the CRISPR-Cas system has been developed into several molecular biology tools, e.g., for genome editing or transcription regulation in bacteria, eukaryotes, and in an even greater number, in archaea [50]. This approach to down-regulate genes in archaea was revealed to be crucial to evaluate gene functions. In vivo experiments were firstly carried out in *H. volcanii*, in which the CRISPR-Cas system was used to inhibit gene expression [50]. Furthermore, the hyperthermophilic euryarchaeon *Pyrococcus furiosus* and *Sulfolobaceae* have been used as models for the study of CRISPR-Cas systems, making them the most explored microorganism among hyperthermophilic archaea [54,55,56]. For example, the in vivo type III-mediated degradation of a viral mRNA in an archaeon was shown firstly in *S. solfataricus* [57], and many efforts have been made to modify the hyperthermophilic archaea’s native CRISPR type III system to silence genes in *Saccharolobus solfataricus* and in the closely related strain *S. islandicus*, leading to the silencing of silence genes involved in cell division (*cdvA*), RNA metabolism (*smAP2*), and transcription (*rpo8*) [58]. In addition, a natural CRISPR type III system was achieved to post-transcriptionally silence host genes, such as α-amylase gene in *S. solfataricus* and *S. acidocaldarius* [54] and the reverse gyrase *TopR1* in *S. islandicus* [59]. Besides some non-essential genes that could be silenced to nearly 100%, type III-mediated knockdown was used on essential genes belonging to cell division, transcription, cell wall biosynthesis, and translation [58,60], allowing for the functional characterization of the relevant genes. This technology has also been used to silence the expression of the S-layer anchor gene *slaB*, allowing it to demonstrate its involvement in cell division and virus infection and to shed some light on the function of the *aIF5A* gene in *S. solfataricus* [60].

### 2.2. Archaea as a Model System of Replication, Transcription, and Regulation of Gene Expression

As mentioned before, the development of the genetic tools for archaea has made it possible to elucidate the role of many of the actors involved in their mechanisms of replication, transcription, and translation, and to compare them with the bacterial and eukaryotic ones. It is outside the scope of this review to report all the studies present in the literature, and only some of the most interesting examples will be reported, those which have made it possible to significantly progress studies of the basic molecular mechanisms of archaea.

#### 2.2.1. DNA Replication and Repair

Bacteria and archaea have a similar genome architecture; however, one of the most intriguing and unexpected findings achieved utilizing knockout studies was the discovery that certain archaeal species do not require a conventional conserved replication origin. It has been discovered that in some archaea, DNA replication can start from a single or multiple origins with a mechanism that is similar to that of eukaryotic cells, involving several origins of replication and proteins similar to the eukaryal initiation proteins Orc1/Cdc6 [61]. Additionally, gene knockout studies have revealed that these origins are not necessary for cell viability in some archaeal species. In the hyperthermophilic euryarchaeon *T. kodakarensis*, it has been shown that the deletion of the putative conserved replication origin does not affect the viability of the organism, suggesting a random DNA replication initiation. Similarly, it was shown that the Orc1/Cdc6 gene can also be deleted in those species [62]. The archaeal primase was formerly assumed to be DnaG, a homolog of the bacterial primase found in the majority of archaeal genomes. Genetic analyses revealed that DnaG is not the archaeal primase. In fact, while DnaG can be knocked out [63] the two-subunit complex of archaeal primase cannot and are both essential for cell viability [13].

A critical protein involved in the initiation of DNA replication is the helicase that unwinds duplex DNA to drive replication forks. In eukaryotic cells, it consists of the heterohexameric minichromosome maintenance (MCM) protein complex, which comprises six subunits (*Mcm2*–7) [64]. In archaea, there are numerous MCM homologous chromosomal genes in several species. The investigation of the function of these genes was made possible by the development of the genetic manipulation system in the hyperthermophilic euryarchaeon *T. kodakarensis* [34]. Gene disruption tests for each MCM gene showed that MCM3 could not be knocked out, unlike the other two MCM genes, strongly indicating that MCM3 is the main helicase in the DNA replication process in *T. kodakarensis* [65]. Unlike eukaryotes, in which MCM helicase requires the association of two accessory factors, the tetrameric GINS (GINS is derived from the Japanese go-ichi-ni-san, meaning 5-1-2-3, as the subunits are Sld5, Psf1, Psf2, and Psf3) complex and the Cdc45 protein, to function, the archaeal one is active, in vitro, without other components [66]. However, it has been demonstrated that not only MCM but also GINS proteins are essential for archaea [67]. The development of genetic tools allowed also for characterizing the archaeal DNA polymerases. DNA polymerase enzymes are classified in seven families, A, B, C, D, E, X, and Y. There are five DNA polymerases in *E. coli*. PolI, PolII, and PolIII, all belonging to different families (A, B, or C), while the DNA polymerases for trans-lesion synthesis, PolIV and PolV, are classified as members of family Y. In eukaryotes, trans-lesion DNA polymerases are members of family Y, while replicative DNA polymerases, Pol-alpha, Pol-beta, and Pol-gamma, are members of family B. Intriguingly, the distribution of polymerases within the archaea follows a phylogenetic distribution. Family B polymerases are present in all archaea, whereas family D polymerase is absent in crenarchaeal species [68]. Therefore, it was postulated that all archaea’s PolB family members are in charge of chromosomal replication [69]. However, the genes encoding for the two PolD subunits are frequently found in operons with other replication enzymes and are located near to the replication origin in many archaeal species [70]. Therefore, it was suggested that PolD may also be active at the archaeal replication fork similarly to eukarya, where Pol-delta duplicates the lagging strand, while Pol-epsilon duplicates the leading strand [71]. Gene knockouts of archaeal DNA polymerases were fundamental to shed some light on the role of the two archaeal polymerases. Studies on *T. kodakarensis* revealed that while PolD was necessary for cell survival, PolB was not, suggesting that PolD could perform both leading and lagging strand synthesis in the absence of PolB [72].

Numerous genetic studies focusing on genes involved in DNA replication and DNA topological maintenance have been reported on *Sulfolobus islandicus*. The deletion of the topoisomerase III gene determined the abnormal chromosome distribution, cell size, and gene transcription, but the cells are still viable, suggesting that this enzyme is crucial for maintaining the DNA architecture for gene transcription and chromosome segregation [73], and another study demonstrated the essentiality of proliferating cell nuclear antigens (PCNA) [74].

Genetic knockout techniques were also used to investigate archaeal DNA repair genes. In this context, successful knockout experiments have been performed on *H. volcanii*, leading to the silencing of *radA*, which acts as a catalyst for strand exchanges in homologous recombination [75]. The Δ*radA* strain showed a higher sensitivity to DNA-damaging agents and an important deficiency in homologous recombination. In addition, researchers were able to perform knockouts of several genes involved in homologous recombination, such as *radA*, *rad50*, and *mre11*, suggesting that homologous recombination is essential for the survival of *T. kodakarensis* and not *H. volcanii* [76]. Several other genes responsible for DNA repair, such as *Hjm*, a *RecQ-like* helicase, *Hjc*, a structure-specific endonuclease, *Hef*, a helicase/nuclease that supports stalled replication forks, and the endonuclease *RNaseH2*, which cleaves at rNMP:dNMP junctions, have been successfully silenced in *T. kodakarensis* [76]. The importance of homologous recombination has also been demonstrated in Sulfolobus, as it has been found that the genes radA, hjm, rad50, mre11, herA, and nurA are necessary for cell viability [77]. Moreover, all genes involved in the NER system in archaea have been examined in Sulfolobus via gene deletion, including the DNA helicase-encoding genes *xpd*, *xpb1*, and *xpb2*, as well as the nuclease-encoding genes *xpf* and xpg/fen1, and only the last one has been found to be necessary [30]. By contrast, DNA repair abilities have not been compromised by the gene deletion of the xp genes encoding for helicases in *S. islandicus* [78], suggesting that these eukaryotic NER-like proteins may play another role aside from DNA repair in this archaeon.

#### 2.2.2. Transcription

The basal archaeal transcription machinery represents an evolutionarily ancient core of the eukaryal RNA polymerase II system that includes RNA polymerase subunits, basal transcription initiation and elongation factors, and central promoter elements. Although regulatory basal transcription factors (TF) that influence the initiation and direct access of basal TFs to specific genomic loci in archaea tend to be bacterial-like, archaeal genomes encode eukaryotic-like promoter elements and basal TFs to the exclusion of bacteria [79]. The mechanisms of initiation have been characterized in great detail in vitro. The basal transcription factors TATA binding protein (TBP) and Transcription Factor B (TFB) bind to their respective promoter elements (TATA box and BRE, respectively) and sequester the RNA polymerase to form the minimal pre-initiation complex. A third transcription initiation factor TFE binds to the polymerase to form the full pre-initiation complex and facilitates DNA melting, leading to the formation of the open complex. TFE stimulates transcription initiation in vitro from some promoters and under sub-optimal conditions, but it is not essential in vitro. The transcription elongation complex also corresponds to an evolutionarily ancient complex comprising homologs of a subset of elongation factors, while transcription termination occurs via intrinsic or factor-dependent mechanisms. In vivo studies have shown that archaea must retain at least one gene encoding TBP and one gene encoding TFB, although many archaeal species encode multiple TBP and TFB isoforms. TFE, although it is not essential in vitro, seems to be essential in vivo, and it is still unclear if this essentiality is due to necessary activities during transcription initiation or some other role during transcription [76]. In *T. kodakarensis*, the role of specific transcription factors has been studied by mean of genetic tools, including those of TFB1/2 [28], RNA polymerase subunits E and F [80], and the switch 3 loop of subunit B [29], and transcriptome analysis after transcription regulator gene deletion allowed for the discovery of regulons and their function [81].

The advance in the development of genetic tools was fundamental to study gene transcription regulation in archaea [82]. Archaea perform gene regulation at different levels, including transcriptional regulation, for which gene-specific transcription regulators play crucial roles for an adequate adaptation to environmental and nutritional changes in order to preserve the survival and fitness of the organism. For example, in *H. salinarum*, seven TFBs and six TBPs are encoded, and genetic manipulations allowed for demonstrating that some genes are not amenable to knockout, given their essential functions in growth, and that each TFB–TBP pair is required for growth under a specific subset of environmental conditions [83]. Furthermore, the development of archaeal genetic tools allowed for determining the function in vivo of several transcriptional regulators such as the Lrp-like transcriptional regulator, the copper-responsive transcription activators [84] in *S. solfataricus*, and the transcription factors involved in iron homeostasis in *H. salinarum* [85]. Genetic studies in *Methanococcus* allowedfor the identification of regulatory factors that govern the expression of genes for hydrogen metabolism and nitrogen assimilation [86].

#### 2.2.3. Translational Recoding

**Figure 3:**
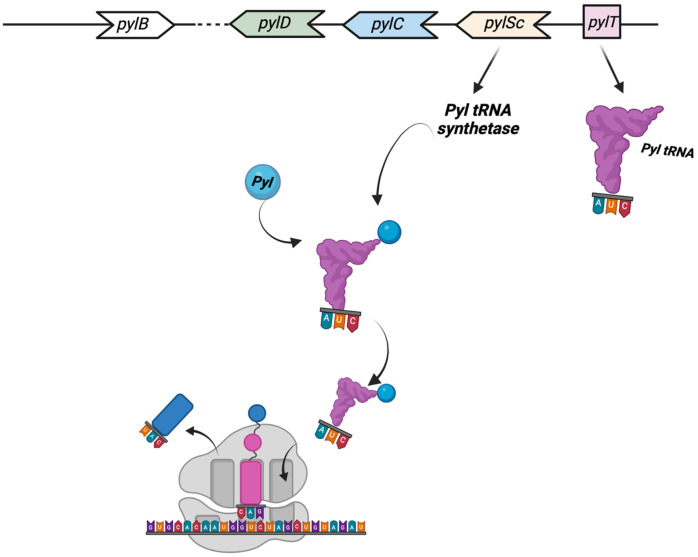
The Pyl insertion system. Pyl, synthesized by *pylB*, *pylD*, *pylC*, is charged on a specific tRNA (encoded by *pylT*) whose anticodon AUC recognizes UAG codons. See text for details. This figure was created with BioRender.com (accessed on 7 December 2022).

One of the most astonishing discoveries in archaea translation was the identification, in 2002, of the 22nd proteinogenic amino acid Pyrrolysine (Pyl) [87,88]. Pyl is a typical l-lysine amino acid to which a pyrrole ring is branched on the lateral chain through an amide bond and is translationally incorporated [89]. The first indication of Pyl’s presence has been reported in several Methanosarcina species, with a total of 21 genes of mono, di-, and trimethylamine methyltransferases (*MtmB*, *MtbB*, and *MttB*, respectively) showing an in-frame amber UAG codon [89]. This unusual and highly specialized amino acid is found in a small number of archaea able to metabolize methylamine as well as a few bacteria. Pyl is found in all methanogen methylamine methyltransferase genes, and in some cases, the readthrough efficiency of the UAG codon is as high as 97%. The five *pyl* genes involved in the biosynthesis and genetic encoding of Pyl are *pylTSBCD* [88], and in most cases, they are organized in an operon-like structure. The enzymes PylB, PylC, and PylD synthesize Pyl from two equivalents of lysine, while *pylT* and *pylS*, encode, respectively, for the tRNAPyl, whose anticodon is complementary to the UAG codon, and the subunit of the tRNAPyl synthetase, which directly esterified Pyl to the 30-hydroxyl of tRNAPyl (Figure 3) [89].

The *pyl* genes have been discovered in the genomes of bacteria and archaea, and they are typically grouped together with other genes involved in the methylamine metabolism and methylamine methyltransferases [90]. Interestingly, the presence of genetic tools for *Methanosarcina acetivorans* allowed for getting more information on this mechanism of translational regulation in vivo. The *M. acetivorans* strain knocked out of the *pylT* gene (encoding tRNAPyl), as the wild type, was able to grow on methanol or acetate, but not on methylamine, nor monomethylamine. In fact, Δ*ppylT* phenotype, whose strain resulted in being unable to decode UAG codons as Pyl, revealed a deficiency in the methylamine metabolism, but also a non-completely compromised growth on other substrates, suggesting that the deletion of the *pylT* gene is conditionally lethal, relying on the growth substrate, and that a comprehensive genetic study of UAG translation as Pyl is possible [91]. The genetic code expansion by the insertion of non-canonical aminoacids is becoming a powerful tool in synthetic biology, for example, to drive the incorporation in a specific site of non-canonical amino acids into a protein [92]. The insertion of Pyl at in-frame UAG codons in specific genes is one of the mechanisms of regulations of translation, occurring during translational elongation or termination steps, which is globally known as *recoding* [93,94], that are frequently in competition with standard decoding and have important roles in gene expression regulation [95]. Translational recoding comprises events that occur during translation and has been observed in all domains of life; these events are known as stop codon readthrough, programmed ± 1 frameshifting, and ribosome bypassing. These events regulate protein expression at the translational level, and their mechanisms are well known and characterized in viruses, bacteria, and eukaryotes. In archaea, it was demonstrated and studied that translational recoding regulates the decoding of selenocysteine and pyrrolysine, the 21st and the 22nd amino acids, and one case of programmed −1 frameshifting has been reported so far in Saccharolobus solfataricus P2 [94,96,97,98,99].

The twenty-first amino acid is selenocysteine (Sec), an amino acid containing the essential micronutrient selenium, that is incorporated during translation into proteins of bacteria, eukarya, and archaea [94]. In the presence of *cis*-specific regulation signals, Sec is inserted in correspondence of UGA stop codons; in this case, translating ribosomes are loaded with a specific Sec-tRNA and promote the insertion of a Sec residue in that point. In fact, a Sec-specific elongation factor (SelB) replaces the common EF-Tu specifically for the translation of Sec UGA codons and recruits the particular Sec-tRNA in response to those signals [94]. With the exception of selenophosphate synthetase (SPS), which is involved in Sec biosynthesis, selenoproteins are frequently enzymes with oxidoreductase functions in which Sec is the catalytic redox active site. While, in bacteria, they are involved in redox homeostasis, electron transport/energy metabolism, compound detoxification, and oxidative protein folding, in archaea, they are involved in methanogenesis [100]. In all the organisms, Sec is synthesized in a tRNA-bound fashion but with some differences [101]. The selenoprotein gene transcripts in cis contain unique signals that activate the insertion process. These signals are RNA structures known as SECIS (SElenoCysteine Insertion Sequence) elements, which cause the specific elongation factor SelB to take the place of the default EF-Tu and to recruit the Sec-tRNA, thereby facilitating the insertion of Sec residues in a particular UGA. The phenotype of a mutant strain with a deletion in the selenocysteine-specific translation factor *selB* gene resulted in an outcome similar to that of the *M. acetivorans* strain with the *pylT* gene deleted (see above) [102]. In particular, the *selB* mutant survived when producing methane from hydrogen and CO_2_, and not when using formate, as *selB* deficiency was associated with decreased activity of the formate dehydrogenase. Sec is predicted to be present in both *M. maripaludis* formate dehydrogenases. The loss of the UAG-dependent *pylT* translation led to the formation of a mutant that, as in *M. acetivorans*, could only survive on methylamine or methanol, correlating with the loss of MMA methyltransferase production. These findings suggest that 21st and 22nd amino acids both contributed significantly to the narrow substrate spectrum diversification of the methanogenic archaea [88]. From a biotechnological point of view, recoding stop codons in the genetic code to introduce non-natural amino acids by using wild-type or engineered aaRS/tRNA pairs from organisms that naturally possess an expanded genetic code, such as pyrolysin aaRS/tRNA from *M. barkeri*, is now used to introduce new functions into proteins and enzymes. To date, well over 100 different unnatural amino acids have been successfully encoded into proteins, providing a variety of functions such as spectroscopic labels, reactive groups for click chemistry, chemical warheads, coordination sites for metals, and more, and the first commercial applications are emerging [88].

**Figure 4:**
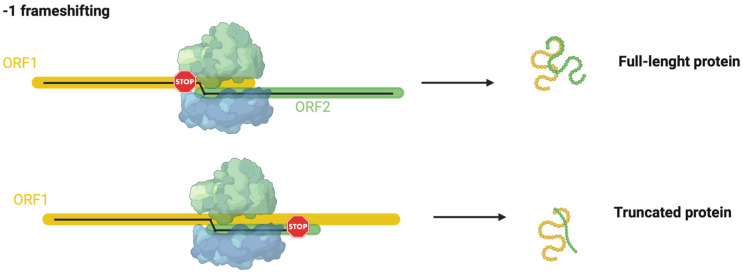
−1 frameshifting mechanisms summarized. ORF1 is underlined in yellow. This figure was created with BioRender.com (accessed on 7 December 2022).

Programmed −1 frameshifting (−1PRF) is another recoding mechanism demonstrated in archaea [8]. In this mechanism, during translation elongation, ribosome slippage to an alternative reading frame of mRNA, leading, in a regulated manner, to the shifting in the +1 or −1 direction [93,94]. The slippage can determine (1) the production of an extended, functional polypeptide from an alternative reading frame with efficiencies varying from very low to as high as 80%, and (2) the production of a non-functional polypeptide as the ribosome encounters a stop codon in the new reading frame (Figure 4) [94,103,104].

In archaea, only one case of −1PRF has been reported to date [98,104]. In the thermoacidophilic archaeon *S. solfataricus* strain P2, the *fucA1* gene was found to be organized in two open reading frames (ORFs) SSO11867 and SSO3060 of 81 and 426 amino acids, respectively, which are separated by a −1 frameshifting in a 40-base overlap. These ORFs encode, respectively, for the N- and C-terminal part of a α-l-fucosidase enzyme. The overlapping region displays distinctive signals found in genes expressed by −1PRF in bacteria and eukaryotes, such as an heptanucleotide A-AAA-AAT, followed by a putative stem and loop secondary structure, and rare codons (CAC) in tandem, which help to facilitate the sliding of the ribosomes. A functional enzyme has been obtained by producing a full-length gene, named *framefucA*, restoring a single reading frame between the two ORFs by inserting in the position predicted by specific −1PRF site-directed mutations in the *fucA1* gene [96]. The *framefucA* mutant produced a fully functional α-l-fucosidase, named Ssα-fuc, which was thermophilic, thermostable, and had an unusual nonameric structure [105,106,107]. The interrupted gene *fucA1* is translated by −1PRF in both *E. coli* and *S. solfataricus*, producing a full-length protein showing for the first time that this kind of recoding is present in archaea [98]. Moreover, only the wild-type slippery sequence in *S. solfataricus* resulted in being functional, as shown by the in vitro translation of *fucA1* and the mutant gene in the slippery sequence [98]. The majority of the studies performed to demonstrate this mechanism of gene expression in *S. solfataricus* were performed by the in vitro translation and biochemical characterization of mutants in the slippery sequence recombinantly expressed in *E. coli*. Therefore, other studies have recently been reported suggesting the possible function in vivo of this gene [7,99]; however, these results need to be further confirmed. Although this phenomenon might have some implications for the physiology and adaptation of life in harsh environments and further possible translational recoding events have been suggested in other archaeal species, this area of research is currently understudied. There is a pressing need to study these recoding episodes in archaea by applying ad hoc genetic tools.

## 3. Discovery and Biotechnological Applications of Archaeal Enzymes

With the growth and development of green biotechnologies, the interest in the use of enzymes as a strategy towards attaining a biobased economy has increased considerably [108]. Enzymes are greener and sustainable substitutes to the use of pollutant chemicals for industrial processes. Enzyme-catalyzed reactions are specific and produce less by-products, and any by-products are of low toxicity [109]. Enzymes are crucial tools in a variety of industrial markets, including those for biofuels, leather, pulp and paper, textiles, animal feed, food and beverage, and detergents. The use of enzymes is also expanding in industries such as medicines, research and development, and diagnostics. The global enzymes market was valued at $6.4 billion in 2021 and is projected to reach $8.7 billion in 2026 at a compound annual growth rate (CAGR) of 6.3% from 2020 to 2026 [110].

The majority of modern industrial processes are carried out in severe conditions with high or low temperatures, acidic or basic pHs, and high salinity. As a result, (hyper)thermophilic enzymes have some advantages over their mesophilic counterparts from an industrial perspective, such as their ability to function at high temperatures, extreme pH levels, high substrate concentrations, high pressure, and in the presence of organic solvents and denaturing agents. According to analysis by Fact.MR (https://www.factmr.com/; accessed on 19 October 2022), enzymes active on carbohydrates account for 44% of the global industrial enzymes market. Glycoside hydrolases (GH) are a large class of enzymes, which catalyze the hydrolysis of the carbohydrates. Based on their amino acid sequences, they are classified in the CAZy database (http://www.cazy.org; accessed on 21 November 2022) in 173 families that show conserved catalytic mechanism, structure, and active site residues, but differ from each other in substrate specificity.

This section of the review will focus on the discovery and biotechnological applications of glycoside hydrolases (GH) from hyperthermophilic archaea.

### 3.1. Enzyme Discovery

**Figure 5:**
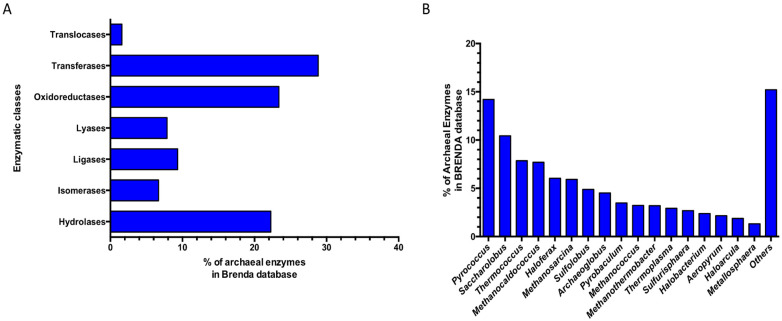
Distribution of the archaeal enzymes in the BRENDA database. (**A**) Relative abundances in enzymatic classes. (**B**) Relative abundances among the archaeal genera. Others: genera with abundance < 1%.

Since 1987 the BRENDA database (www.brenda-enzymes.org; accessed on 21 November 2022) has represented the main database related to the classification of enzymatic activities reported in the literature, including taxonomic references and sequence identifiers for enzymes [111]. A search updated in November 2022 reports in the BRENDA database, compared to 33,735 enzymes of bacterial origin, only 3938 enzymes from archaea distributed in all the main enzyme classes: Oxidoreductases (EC 1), Transferases (EC 2), Hydrolases (EC 3), Lyases (EC 4), Isomerases (EC 5), Ligases (EC 6), and Translocases (EC 7). In particular, among these, the Transferases (28.8%), Oxidoreductases (23.3%), and the Hydrolases (22.3%) represent the most abundant classes characterized in the Archaea domain (Figure 5A).

More in detail, the sub-sub-classes of enzymes discovered and characterized so far in archaea (Table 1) are mainly oxidoreductases, which act on the CH-OH group of donors with NAD^+^ or NADP^+^ as an acceptor (EC 1.1.1.-), followed by glycosidase (EC 3.2.1.-) and nucleotidyltransferases (EC 2.7.7.-) representing 6.58%, 5.23%, and 4.24%, respectively.

An in-depth taxonomic survey of archaeal entries, at the genus level, reveals that these are mainly of an euryarchaeal origin belonging to the classes of Halobacteria, Methanobacteria, Methanococcia, Methanomicrobia, Thermococci, and Thermoplasmata followed by Crenarchaeota (Thermoprotei) (Figure 5B), while only 14 enzymes present in the database come from uncultured microorganisms of archaeal origin.

**Figure 6:**
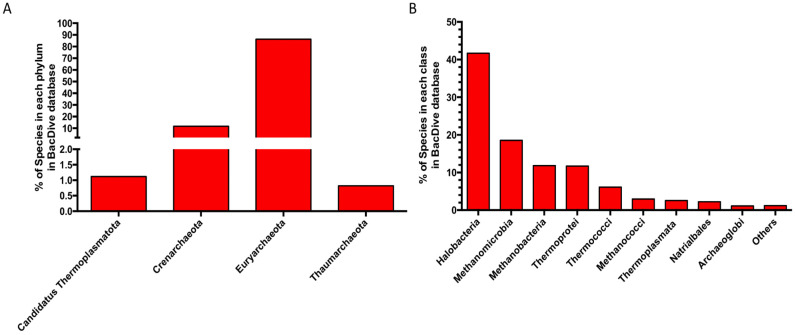
Distribution of the archaeal species in the BacDive metadatabase. (**A**) Phylum level. (**B**) Class level. Others: classes with abundances < 1%.

The considerable difference between the number of archaeal enzymes characterized, compared to those from bacteria, is also evident considering the different number of species belonging to archaea and bacteria isolated and characterized so far to date in BacDive Metadatabase (https://bacdive.dsmz.de/, accessed on 21 November 2022), one of the most complete resources on prokaryotes and containing data on taxonomy, morphology, physiology, cultivation, and isolation [112]. There are 90,100 bacterial species of which the parameters listed above are known, while the entries related to archaeal species are only 981. Among these, most species belong to the phyla Euryarchaeaota (86%) and Crenarchaeota (12%), mainly belonging to the class of Halobacteria (42%) (Figure 6A,B).

**Figure 7:**
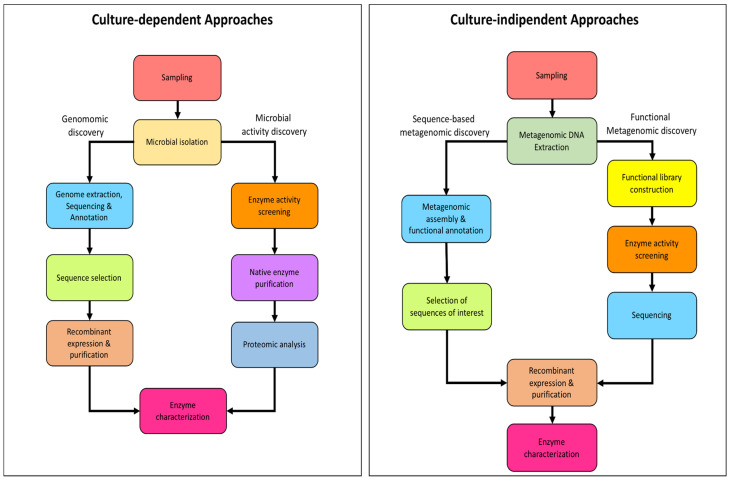
Schematic flowcharts of enzyme discovery approaches.

These differences between bacteria and archaea, in terms of cultivable microorganisms and characterized enzymes, can be explained by considering both the general limits in the isolation of extremophiles, which are mainly archaea [113] with a difficulty to efficiently express and archaeal genes in recombinant form in a bacterial host [114], especially when culture-dependent and -independent approaches are compared in enzyme discovery flowcharts (Figure 7).

The culture-dependent approaches can be summarized in (i) Genomic discovery, and (ii) Microbial activity discovery. These approaches are frequently applied in parallel and bound to the possibility of isolating and growing in the laboratory the microbial strains that will serve as the source of the appropriate enzymatic activity.

For what it concerns Genomic discovery, access to the genome sequence is sufficient. The genomes functionally annotated by bioinformatic analysis allow for the identification of genes encoding for putative enzymes that through recombinant expression can be obtained and subsequently characterized [115]. This approach has allowed for the identification of several archaeal enzymes such as the β-glucosidase/β-xylosidase from *S. solfataricus* belonging to the glycosidase family GH116 [116].

Conversely, the second approach necessarily requires the manipulation and growth in the laboratory of the previously isolated microorganism of interest, which can be extremely challenging in the case of extremophilic archaea. In fact, the first step consists of an activity screening of cell extracts usually by using chromogenic substrates. The enzymatic activities obtained are then analyzed by proteomic analysis to allow for their identification. In this case, the main limitations consist of substrates that can be used, commercially limited to a few classes of molecules. In addition, the levels of expression of endogenous proteins could be too low to obtain reliable results. However, by using this approach, several archaeal enzymes have been successfully identified for the first time and characterized, both in their native and recombinant form [117,118,119,120].

Although, to date, the growth and isolation techniques for archaea have been improved [121], the isolation and growth of extremophiles in laboratory conditions still represent a limit to explore the microbial diversity and to identify their biocatalysts [122] However, the evolution of both Next-Generation DNA Sequencing technologies (NGS) and high-throughput screening platforms has enabled the development and improvement of culture-independent enzyme discovery approaches: sequence-based metagenomics and functional metagenomics (Figure 7). Both approaches have as the only prerequisite the purification of metagenomic DNA (mDNA) from a starting sample and are now widely used, for both the identification of bacterial and archaeal enzymes [123].

The sequence-based enzyme discovery approach involves NGS sequencing of mDNA, its assembly, and then functional annotation by using both public and tailored functional databases generally based on hidden Markov models (HMMs) [121]. Thus, the coding sequences for the enzymes of interest identified in silico can be cloned or used for the preparation of synthetic genes into bacterial expression vectors. Recently, with this approach, it was possible to discover two new enzymatic activities, a β-mannanase/β-1,3-glucanase and a hyperthermophilic β-*N*-acetylglucosaminidase/β-glucosidase originating from a novel unidentified microorganism belonging to the Sulfolobaceae family [9]. In addition, the sequence-based metagenomic approach has also proved powerful for the detailed analysis of the potential catalogue of carbohydrate-active enzymes, allowing for the detailed mapping of archaeal enzymes both in the environmental samples from high-temperature environments and in laboratory samples enriched on lignocellulosic biomasses [8,124,125].

Alongside the sequence-based metagenomic enzyme discovery, high-throughput functional screening is another powerful approach for identifying novel enzymes and assigning gene functions, especially when isolation and microbial cultivation in the laboratory are limited. This method is based on the functional screening of mDNA libraries to identify the expression of new enzymatic activities [121,126]. The libraries are built by cloning fragments of purified mDNA from environmental samples into an empty vector. The size of the mDNA insert depends on the type of vector used and ranges from <10 kb to 200 kb for plasmid vectors and bacterial artificial chromosomes, respectively.

For the functional metagenomic enzyme discovery approach, cosmids and fosmids are the most widely used vectors due to the large size of the mDNA insert they host (~40 kb), the ease of manipulation, and the availability of commercial kits to build and isolate them. In addition, fosmids offer an arabinose-inducible copy control system, which allows the number of copies of the phosphide per cell (usually *E. coli*) to be adjusted, allowing for better protein expression during the activity screening. This latter occurs by measuring enzyme activity on labeled substrates such as chromogenic and fluorogenic substrates [127,128], or alternatively, as for the discovery of archaeal endoglycosidases, on natural substrates such as cellulose or starch [129,130]. Only once the activity has been identified will it be necessary to proceed with the vector sequencing for the identification of the associated sequence. Then, the gene can be expressed in a recombinant form on a larger scale and subsequently characterized.

The main drawback that can compromise the functional metagenomics approach is the selection of the expression host. Although commonly used *E. coli* strains have relaxed requirements for promoter recognition and translation initiation, some genes from environmental samples may not be efficiently expressed due to active enzyme toxicity, or more generally, due to differences in codon-usage, post-translational modifications, protein folding, and transcription and/or translation initiation signals [114].

Despite these limitations, culture-independent approaches represent a powerful tool for enzyme discovery compared to the culture-dependent counterpart, especially when these are applied in a complementary way. In fact, the combination of the two approaches allows for the discovery of novel enzymes without the limitation of the annotation by homology by using enzymatic classes already identified [131].

### 3.2. Archaea Enzymes and Their Applications

The utilization of thermoacidophilic archaea and their enzymes offers certain benefits for industrial biotechnology [5]; indeed, at high temperatures, reaction rates increase and substrate accessibility improves. The energy input for cooling steps in bioreactors and the relative costs can be reduced. Furthermore, microbial contamination is negligible, so the use of antibiotics can be limited [6,132].

However, the cultivation of these microorganisms on a large scale is difficult, and the growth yields are typically low. To overcome these problems, (hyper)thermophilic enzymes are produced in mesophilic hosts. The heterologous production allows for obtaining much faster growth rates, highly efficient expression, and facilitated downstream processing of thermostable proteins, since the host proteins can be rapidly removed via heat precipitation. Nevertheless, the production yield of archaeal proteins could be low due to differences in the expression and folding machinery [6,133]; extremophilic archaea as metabolic engineering platforms through the evolution of molecular genetics tools for these microorganisms can be used as also described in the previous paragraphs. Well-developed genetic toolboxes exist for the euryarchaeal *T. kodakarensis*, *P. furiosus*, *H. volcanii*, and for methanogenic archaea [15]. In addition, for the genus Sulfolobus, a large number of genetic systems is established [15], although no industrial process utilizing *Sulfolobus* spp. has been developed yet. As a result, enzymes from thermoacidophilic Sulfolobales are still mainly produced in recombinant form in mesophilic hosts such as *E. coli* [7,116,117,118,125].

Many different hyperthermophilic archaeal GHs has been identified and characterized [134,135]; they mainly include starch and (hemi)cellulose, since biofuel production processes are typically carried out at high temperatures and a low pH, but also because chitin-degrading enzymes exhibit promising applications in pharmaceutical processes [136,137].

#### 3.2.1. Starch-Degrading Enzymes

Many economically significant crops, including wheat, rice, maize, tapioca, and potatoes, generate starch as a key storage product. Enzymatic hydrolysis of starch requires the concerted action of many glycoside hydrolases: α- and β-amylase, also known as endo-amylases, producing oligosaccharides; the exo-amylases glucoamylases; and α-glucosidases producing glucose [138].

α-Amylases (endo-1,4-α-d-glucan glucohydrolase, EC 3.2.1.1) are among the popular enzymes used industrially to cleave the 1,4-α-d-glucosidic linkages between glucose units in the linear amylose chain obtained from starch [139]. They belong to families GH13, GH57, and GH119 in the CAZy database [140]. Only members from families GH13 and GH57 belonging to *Pyrococcus* sp. *ST04*, *Pyrococcus woesei*, *Thermococcussp. HJ21, Saccharolobus solfataricus*, and the methanogenic archaeaon *Methanococcus jannaschii* have been characterized [141].

Commercially, thermostable amylases occupy almost 30% of the enzyme market [121]. Bioconversion of starch to sugars using enzymes requires three steps: gelatinization, liquefaction, and saccharification. Gelatinization that dissolves starch granules is generally carried out at 110 °C; thus, most of the starch enzymatic conversion is carried out with thermostable α-amylases that are favored for their productivity and economic significance [142].

As exoamylases, α-glucosidases (EC 3.2.1.20) break every α-1,4-glycosidic bond from the terminal non-reducing end of smaller oligosaccharides of the starch, generating glucose [141]. These enzymes are involved in the last step of starch degradation. Although α-glucosidases belong to families GH4, GH13, GH31, GH63, GH76 and GH122 [140], only archaeal members of the GH31 and GH122 families have been characterized. Most of them are hyperthermophilic archaea from the genera *Pyrococcus* and *Thermococcus* [143,144]. Additionally, there are α-glucosidases from the acidophilic archaeon *Ferroplasma acidophilum* strain Y [145] and from the thermoacidophilic archaeaon *Sulfolobus* (Park et al. 2013) and Picrophilus [146].

#### 3.2.2. Cellulose-Degrading Enzymes

Cellulose is a major component of the plant cell wall and hence the most abundant polymer on Earth. About 1.5 trillion tons of cellulose is produced annually [147]. Due to its complex structure, cellulose hydrolysis requires a concerted action of cellulases: β-endoglucanase (also known as Cellulase, EC 3.2.1.4) that cleave the polymer to produce short oligosaccharides followed by the simultaneous action of 1,4 β-cellobiohydrolase (EC 3.2.1.91) and β-glucosidase (EC 3.2.1.21), which hydrolyzes it to glucose [148].

Cellulases (EC 3.2.1.4) belong to 14 different GH families [140]. Among the characterized cellulases, only members of families GH5 and GH12 come from hyperthermophilic archaea [119,135,149,150]. In the last decades, an immense increase in cellulases’ applications in biofuel and the pulp and paper industries has been observed [142] as well as in detergent, textile, animal feed, fruit juice industries, and in bioremediation [142]. Indeed, as reported in the global cellulase (Cas 9012-54-8) market research report 2022, the global cellulase market is estimated at US$ 1621 Million, with a growing CAGR of 6.9% from 2022 to 2032.

In pulp industries, endoglucanases can reduce the pulp viscosity with a lower degree of hydrolysis, and the use of these enzymes brought an energy savings of nearly 20–40% [151]. Thanks to the strong resistance to anionic surfactants, oxidizing agents, and thermostability, cellulases can also be promising to be used in laundry detergent together with proteases [152]. Further, in bioremediation, cellulases can be potentially used to extract cellulose from hypersaline effluent [153]. There are several potential industrial applications of cellulases, the main commercial potential of using these enzymes in second generation biorefineries. Cellulases are able to convert lignocellulosic biomasses into glucose which can be utilized in bioethanol production [154]. It is envisaged that cellulases may become the most produced industrial enzyme if ethanol from lignocellulosic biomass becomes a major transportation fuel [155].

#### 3.2.3. Xylan-Degrading Enzymes

The natural biomass of lignocellulose finds potential as an alternate energy source exhibiting the possibility of replacing the fossil sources. Lignocellulose is mainly composed by cellulose (40%), hemicellulose (33%), and lignin (23%) [156]. Xylan is the main constituent of the hemicellulosic compounds that accounts for one-third of total organic carbon present on earth [157]. Xylan-degrading enzymes groups a plethora of enzymes that include endoxylanases, β-xylosidase, arabinofuranosidase, and acetyl-xylan esterase [158]. Endoxylanases (or xylanases, EC: 3.2.1.8) cleave the β-glycosidic bonds of the xylan backbone and release xylooligosaccharides as a product, while the β-xylosidases (EC: 3.2.1.37) act on xylobiose or other xylooligosaccharides to generate monomeric sugar xylose as the final product. Arabinofuranosidases (EC: 3.2.1.55) and acetyl xylan esterases (EC: 3.1.1.72) attack on side chains of heterogeneous xylan substrate and assist xylanases and β-xylosidases for the complete degradation of xylan [158]. Xylanases (EC: 3.2.1.8) represent the chief enzyme of hemicellulases and find large employment in the second generation biorefineries [159]. Xylanase (EC: 3.2.1.8) are classified into nine families (GH5, GH8, GH10, GH11, GH30, GH43, GH51, GH98, GH141) in the CAZy database, most of them in the families GH10 and GH11 [140,160].

Over biofuel production, thermostable xylanases find application in many industrial fields: in the paper industry, xylanases could be employed in the bio-bleaching of pulp samples to reduce the use of chlorine derivatives for achieving an optimal brightness of the paper [160]; in the pharmaceutical industry, xylanases produce the prebiotic xylooligosaccharides from xylan degradation. In addition, xylanase treatment improves the quality of bread by reducing the staling rate and increases the shelf life [161], as well as reduces the turbidity in fruits and vegetable juices.

#### 3.2.4. Chitin-Degrading Enzymes

Chitin is the second most abundant polysaccharide in the ecosystem after cellulose. It composes the majority of the components of fungal cell walls, as well as the exoskeleton, stomach lining, and crustacean shells [162,163]. Chitinases (3.2.1.14) play a role in the random endo-hydrolysis of *N*-acetyl-β-d-glucosaminide (14)-β-linkages in chitin and chitodextrins [142]. They belong to the families GH18, GH19, GH23 and GH48 in the CAZy database [140]. Among these families, only a few members of chitinase from family GH18 coming from thermophilic archaea have been characterized: PF-ChiA from *Pyrococcus furiosus* [164], Tk-ChiA from *Thermococcus kodakarensis* [165], and Tc-ChiD from *Thermococcus chitinophagus* [166].

Chitinases are useful in forming chito-oligosaccharides, known to possess antitumoral, antimicrobial, immunomodulatory, antioxidant, and anti-inflammatory properties. This makes them of great interest in the food and pharmaceutical industries [167]. In addition, chitinases find application in the biocontrol of fungal phytopathogens and invasive pests affecting crops [168], as well as for the bioremediation in the aquatic ecosystem. In fact, chitin deposits made up of many billion tons of marine trash exist (for example, from shellfish) [169]. These marine wastes could be regulated by a valuable thermophilic enzymatic system to eco-friendlily degrade chitin by avoiding thermochemical processes [170].

## 4. Conclusions

Archaea have gained a lot of attention during the past years as valuable model systems for molecular biology and biotechnology. Nowadays, methanogens, halophiles, thermophilic euryarchaeota, and crenarchaeota are the four groups of archaea for which efficient genetic tools have been established, making them interesting model systems for the elucidation of the basal molecular mechanisms in this domain of life and genetic evolution studies. Among them, thermophiles have been used to explore a variety of aspects of archaeal biology, including cellular responses to stress, DNA replication and repair, transcription and its regulation, and carbon and energy metabolism. Moreover, thermophilic archaea represent a potentially valuable source of new enzymes of potential biotechnological interest and can be used as cell factories to perform microbial processes under extremely hostile conditions.

## Figures and Tables

**Table 1:** Distribution of the sub-sub classes of archaea enzymes in the BRENDA database.

Sub-Subclass	Description	Entries in BRENDA Database	% of BRENDA Archaeal Entries
1.1.1.-	Oxidoreductases acting on the CH-OH group of donors with NAD^+^ or NADP^+^ as acceptor	259	6.58
3.2.1.-	Glycosidases	206	5.23
2.7.7.-	Nucleotidyltransferases	167	4.24
2.1.1.-	Methyltransferases	151	3.83
2.7.1.-	Phosphotransferases with an alcohol group as acceptor	151	3.83
6.1.1.-	Ligases forming aminoacyl-tRNA and related compounds	112	2.84
2.5.1.-	Transferases active on alkyl or aryl groups, other than methyl	98	2.49
4.2.1.-	Hydro-lyases	97	2.46
1.2.7.-	Oxidoreductases with an iron-sulfur protein as acceptor	78	1.98
4.1.1.-	Carboxy-lyases	74	1.88
2.4.99.-	Glycosyltransferases transferring other glycosyl groups	72	1.83
3.1.1.-	Carboxylic-ester hydrolases	68	1.73
2.4.1.-	Hexosyltransferases	64	1.63
3.6.4.-	Hydrolases acting on acid anhydrides to facilitate cellular and subcellular movement	63	1.60
1.2.1.-	Oxidoreductases acting on the aldehyde or oxo group of donors with NAD^+^ or NADP^+^ as acceptor	60	1.52
2.3.1.-	Acyltransferases transferring groups other than aminoacyl groups	57	1.45
3.1.3.-	Phosphoric-monoester hydrolases	54	1.37
6.2.1.-	Acid-thiol ligases	53	1.35
2.6.1.-	Transaminases	52	1.32
1.4.1.-	Oxidoreductases Acting on the CH-NH_2_ group of donors with NAD^+^ or NADP^+^ as acceptor	51	1.30
2.4.2.-	Pentosyltransferases	51	1.30
4.1.2.-	Aldehyde-lyases	51	1.30
6.5.1.-	Ligases that form phosphoric-ester bonds	51	1.30
5.6.2.-	Enzymes altering nucleic acid conformation	49	1.24
3.5.4.-	Hydrolases	48	1.22
3.5.1.-	Acting on carbon-nitrogen bonds, other than peptide bonds in cyclic amidines	46	1.17
6.3.4.-	Ligases Forming carbon-nitrogen bonds other carbon-nitrogen ligases	45	1.14
3.6.1.-	Hydrolases acting on acid anhydrides in phosphorus-containing anhydrides	44	1.12
5.3.1.-	Intramolecular oxidoreductases Interconverting aldoses and ketoses, and related compounds	44	1.12
2.7.4.-	Phosphotransferases with a phosphate group as acceptor	43	1.09
3.4.21.-	Serine endopeptidases	42	1.07
2.8.4.-	Transferases transferring alkylthio groups	41	1.04
3.1.26.-	Endoribonucleases producing 5′-phosphomonoesters	41	1.04
6.3.2.-	Peptide synthases	40	1.02
Others	Sub-sub classes with relative abundance < 1%	1315	33.39

## Data Availability

Not applicable.
